# Unveiling Potential of Gallium Ferrite (GaFeO_3_) as an Anode Material for Lithium-Ion Batteries

**DOI:** 10.1021/acsomega.4c05437

**Published:** 2024-09-13

**Authors:** Mohan K. Bhattarai, Shweta Shweta, Moses D. Ashie, Shivaraju Guddehalli Chandrappa, Birendra Ale Magar, Bishnu P. Bastakoti, Ubaldo M. Córdova Figueroa, Ram S. Katiyar, Brad R. Weiner, Gerardo Morell

**Affiliations:** † Department of Physics, University of Puerto Rico, San Juan, Puerto Rico 00931, United States; ‡ Department of Chemistry, North Carolina A&T State University, 1601 East Market Street, Greensboro, North Carolina 27411, United States; § Department of Chemical Engineering, University of Puerto Rico, Mayagüez, Puerto Rico 00681, United States; ∥ Department of Chemistry, University of Puerto Rico, San Juan, Puerto Rico 00931, United States

## Abstract

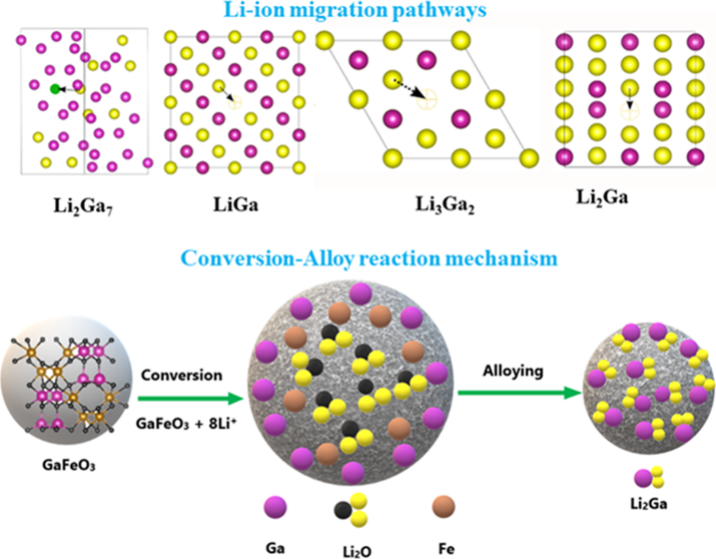

Lithium-ion batteries (LIBs) serve as the backbone of modern technologies with ongoing efforts to enhance their performance and sustainability driving the exploration of new electrode materials. This study introduces a new type of alloy-conversion-based gallium ferrite (GFO: GaFeO_3_) as a potential anode material for Li-ion battery applications. The GFO was synthesized by a one-step mechanochemistry-assisted solid-state method. The powder X-ray diffraction analysis confirms the presence of an orthorhombic phase with the *Pc*2_1_*n* space group. The photoelectron spectroscopy studies reveal the presence of Ga^3+^ and Fe^3+^ oxidation states of gallium and iron atoms in the GFO structure. The GFO was evaluated as an anode material for Li-ion battery applications, displaying a high discharge capacity of ∼887 mA h g^–1^ and retaining a stable capacity of ∼200 mA h g^–1^ over 450 cycles, with a Coulombic efficiency of 99.6 % at a current density of 100 mA g^–1^. Cyclic voltammetry studies confirm an alloy-conversion-based reaction mechanism in the GFO anode. Furthermore, density functional theory studies reveal the reaction mechanism during cycling and Li-ion diffusion pathways in the GFO anode. These results strongly suggest that the GFO could be an alternative anode material in LIBs.

## Introduction

Rechargeable lithium batteries are indispensable in the mobile device sector, electric vehicles, and grid storage applications due to their high reversible capacity and long cycling stability.^1^ However, graphite, despite its widespread use as a negative electrode material in commercial applications, falls short of meeting high specific energy requirements, with its theoretical specific capacity of 372 mA h g^–1^.^2^ As the demand for electrochemical energy storage rapidly advances, the search for electrode materials with high specific capacity and exceptional electrochemical performance has become increasingly urgent.^3−6^ In this context, the potential of GFO as an alternative to graphite is a source of great excitement given its high theoretical specific capacity and stability demonstrated in our study.

In recent years, gallium-based materials have garnered significant interest due to their alloy/dealloy lithium storage mechanism, which can achieve higher capacity in the form of Li_2_Ga alloy, as well as their self-healing reaction mechanism during the cycling process.^7,8^ Indeed, Ga-based materials undergo significant volume changes during the alloy/dealloy process, leading to the pulverization of electrode materials from the current collector. Consequently, this results in rapid capacity decay, poor cycling stability, and low coulomb efficiency.^9^ On the other hand, conversion-based materials, such as FeO,^9^ Fe_2_O_3_,^10^ and CoO,^11^ have also received much attention in the field of Li-ion battery anode materials. Because these materials deliver similarly high capacities as alloy-based anode materials, their large polarization and inferior cycling stability, caused by the conversion reaction during cycling, hinder the commercial application of conversion materials.

To mitigate the aforementioned issues, such as the volume expansion problem in Ga-based materials due to the alloy/dealloy process and the conversion reaction during cycling in conversion-based materials, several approaches can be pursued. Interestingly, there are types of electrode materials that operate via a sequential conversion-alloy reaction, involving an initial conversion followed by a subsequent alloy reaction, such as tin-based materials.^12,13^ Reports indicate that these anode materials demonstrate better cycling life than pure alloy materials, attributed to the formation and presence of lithium oxide (Li_2_O), which can buffer the change in electrode volume change. Interestingly, metal chalcogenides also undergo consecutive conversion and alloying reactions. Recently, Muhammad et al.^14^ reported the Sb_2_Te_3_/CNT composite as an anode material for Na-ion batteries, which delivers a high energy density of 229 Wh kg^–1^ at 0.5C and follows conversion and alloy reactions. Similarly, Jiang et al.^15^ reported SbBiTe_3_/Graphite-based anode materials for K-ion batteries, delivering a remarkable capacity of 202 mA h g^–1^ after prolonged 1000 cycles at current density of 80 mA g^–1^, with Coulombic efficiency (CE) higher than 99 %. Authors attributed that electrodes undergoing reversible conversion-alloying reactions with K-ions could offer much higher capacities than traditional intercalation-based anodes. Motivated by this phenomenon, there is potential to develop more analogous compounds to enrich the conversion-alloy material family and further advance high-energy lithium ion batteries (LIBs).^16^

GFO exhibits a perovskite structure (ABO_3_, where A = Ga atom and B = Fe atom) and possesses multiferroic properties.^17^ In recent years, GFO has emerged as a promising alternative for room-temperature multiferroics. It demonstrates notable spontaneous polarization and ferrimagnetism at ambient temperatures, making it a valuable candidate for spintronic applications.^18^ This material is also appealing as a high-capacity anode for battery applications due to its potential 8-electron conversion-alloy reaction mechanism. Therefore, a high theoretical capacity of 1235 mA h g^–1^ can be expected, nearly tripling the capacity of graphite.^19^ This encouraged us to explore a GFO compound as an anode material for use in rechargeable LIBs. Interestingly, density functional theory (DFT) calculations stand out as a highly potent tool for unveiling new advanced electrode materials, directing efficient structural design, and attaining a profound comprehension of the reaction mechanism and ion diffusion barrier within rechargeable batteries.^20^ Recently, Cui et al.^15^ conducted DFT studies to explore novel anode materials. They succeeded in identifying a series of conversion-alloying reaction-based metal chalcogenides (MCs) K-ion batteries. The DFT calculations showed that as the atomic number of chalcogens increases, the ion diffusion kinetics improve, indicated by lower diffusion barriers and formation energies of ion vacancies, which ultimately influence the diffusion coefficient. Motivated by this work, we also conducted first-principles methods based on DFT to investigate reaction mechanisms during cycling and Li-ion diffusion pathways in the GFO anode.

Notably, GFO has not been extensively studied through either DFT or experimental approaches for electrochemical application. In this work, for the first time, we investigate the electrochemical behavior of this novel alloy-conversion-based anode material for LIBs employing a comprehensive approach that combines DFT-theoretical analysis with experimental validation, emphasizing a new direction for research in the field.

## Materials and Methods and Characterization

### Synthesis of GFO and Coin Cell Assembly

GFO powder was synthesized via a mechanochemistry-assisted solid-state reaction. High-purity oxide materials, including gallium oxide (Ga_2_O_3_: Alfa Aesar; 99.9 %) and iron oxide (Fe_2_O_3_: Alfa Aesar; 99.99 %), were used as starting precursors. The mixture was then subjected to high-energy ball milling using zirconia balls for 12 h to achieve thorough mixing and homogenization of the oxide powders utilizing acetone media as solvent. Following ball milling, the mixture was dried to eliminate the solvent (acetone). The dried mixture was finely ground using a mortar and pestle, ensuring that the resulting powder was well dispersed and uniform. The finely crushed mixture was placed in a closed alumina crucible and then calcined in a Carbolite HTF1700 furnace at 1300 °C for 10 h.

The mixture of GFO and carbon black acetylene (C) was kept in a high-energy ball milling machine for 6 h utilizing zirconia balls. The high-energy environment helps break down the powder particles and achieve a uniform distribution of C within the GFO matrix. A 5 % poly(vinylidene fluoride) (PVDF) solution in NMP solvent was prepared. The anode was fabricated with GFO (70 %), C (20 %), and PVDF (10 %), which was homogeneously mixed by using a mortar and pestle. It was then uniformly coated onto a 9 μm Cu sheet with a doctor blade and dried in a vacuum furnace at 60 °C for 16 h to remove moisture completely. Circular electrodes with a diameter of 10 mm were then cut using a punch. The average active mass (GFO) of the electrodes was 1.3–1.5 mg.

The CR2032 battery case (Land instrument), polypropylene (PP) separator (2400 Celgard), and lithium chips (MSE supplies) as a counter electrode were chosen and assembled within an argon atmosphere glovebox (in a high-purity argon environment with water/oxygen content below 0.1 ppm). An appropriate amount of 1 M lithium hexafluorophosphate (LiPF_6_; MSE supplies) electrolyte with a solvent mixture of ethylene carbonate (EC) and dimethyl carbonate (DMC) in a 1:1 volume ratio was utilized during the assembly process. Subsequently, the assembled batteries were left to rest for 12 h.

### Material Characterization

The crystal phase of the powders at room temperature was examined by employing a powder X-ray diffractometer (Rigaku, Miniflex 600) with Cu Kα radiation. The Horiba-Jobin T64000 spectrometer was used for Raman spectroscopy. Raman spectra were recorded in backscattering geometry. A confocal microscope featuring an 80 × objective with a numerical aperture of 0.9 was used in conjunction with the Raman spectrometer. The small focus spot size was maintained below 3 μm, and the power of the incident laser beam for excitation was set at 2.15 mW. Scanning electron microscopy (SEM) images and energy dispersive spectroscopy (EDS) spectra were acquired using a JEOL JEM-1400 Plus instrument manufactured by JEOL (Peabody, Massachusetts). The microscope was operated at an acceleration voltage of 120 kV (kilovolts) equipped with a LaB_6_ thermionic source. X-ray photoelectron spectroscopy (XPS) analysis was conducted under ultrahigh vacuum (∼ 6 × 10^–9^ Torr) using a Thermo Fisher Scientific instrument (Waltham, MA) source of Al Kα, equipped with a physical electronics 5600ci analyzer to identify the elemental species and chemical composition present on the sample’s surface.

### Electrochemical Characterization

Galvanostatic charge–discharge (GCD) curves were conducted using the multichannel battery test system CT2002A from Landt instrument (Vestal, NY) in a voltage range of 0.01–2.8 V (vs Li/Li^+^) at different current densities. Cyclic voltammetry (CV) tests at various sweep rates (0.1–0.8 mV s^-1^) were performed to assess redox activity, reversibility, and stability during charge–discharge cycles of the battery utilizing an Arbin instrument (MITS Pro8.0). Electrochemical impedance spectroscopy (EIS) measurements were carried out before and after cycling at the open circuit voltage (OCV). A small amplitude AC signal of 10 mV was employed, covering a wide range of frequencies from 0.01 Hz to 1 MHz. These measurements were performed using an interconnected setup between a Gamry potentiostat and an Arbin instrument.

### Computational Method

We applied the first principle computational method based on DFT.^21,22^ All calculations were executed using Quantum ESPRESSO (opEn-Source Package for Research in Electronic Structure, Simulation, and Optimization) electronic structure code.^23^ Our calculation employed plane-wave basis sets with periodic boundary conditions. Spin polarization was incorporated into the computational framework. The plane-wave kinetic energy cutoff was set to 55 Ry for the wave functions and 220 Ry for the charge density. The Brillouin zone was sampled according to the Monkhorst–Pack scheme.^24^ The exchange-correlational functional was approximated using GGA PBE^25,26^ functional. The process of structural optimization was carried out until the interatomic forces were less than 10^–3^ a.u. Diffusion barrier potentials were determined by using the climbing image nudged elastic band (CI-NEB) method implemented in Quantum ESPRESSO.

## Results and Discussion

**Figure 1:**
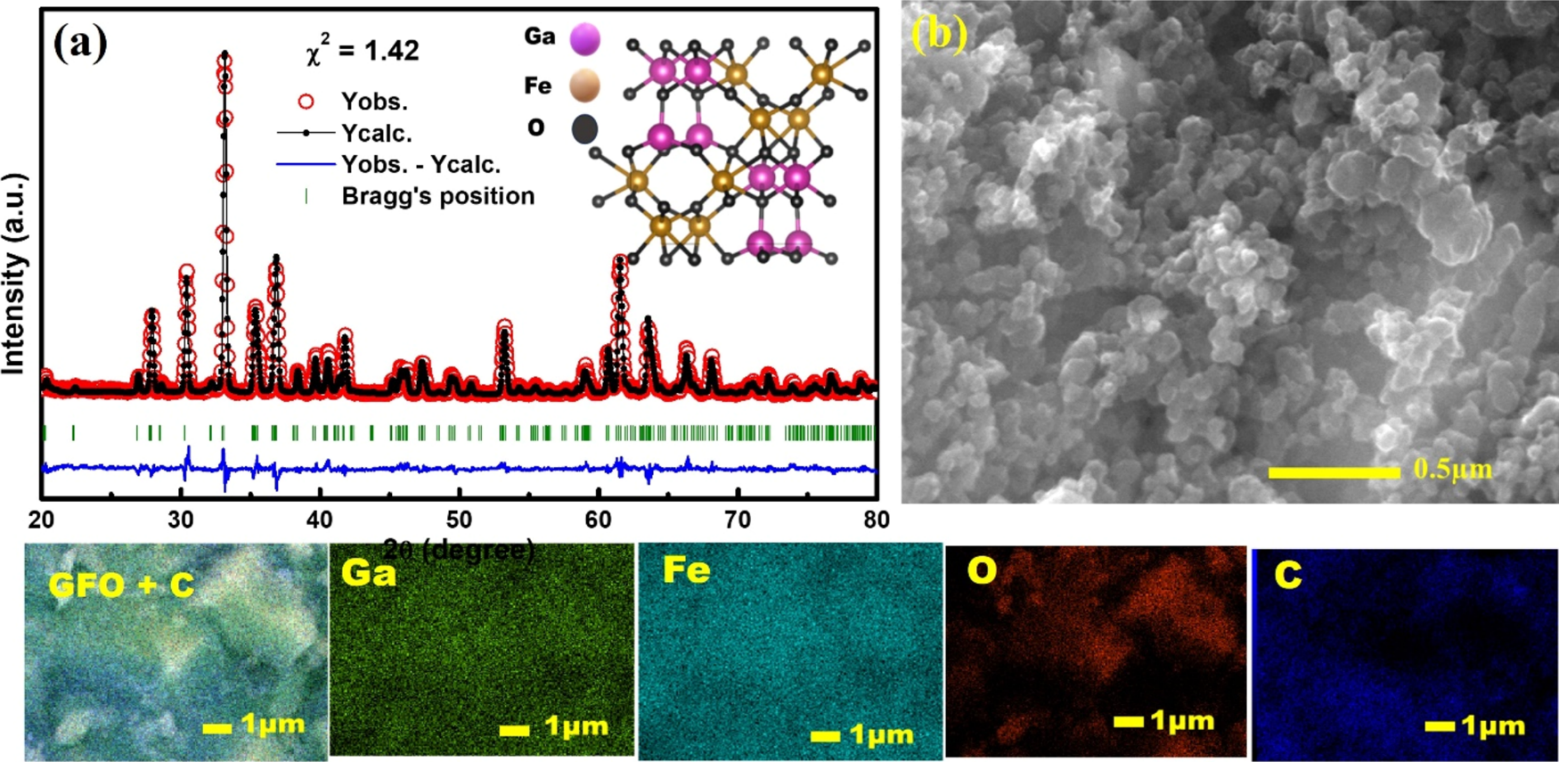
(a) Rietveld refinement of the X-ray diffraction of GFO (Inset: GFO crystal image). (b) SEM micrograph of GFO electrode and EDS elemental mapping of GFO electrode in the second row.

Figure 1a exhibits the Rietveld refinement results of XRD data recorded at room temperature for GFO using the Fullproof suite.^27^ Phase identification for refinement was carried out with CIF file no. 1008838, and profile simulation employed a pseudo-Voigt function. The XRD peak positions obtained from Rietveld refinement accurately were well aligned with the JCPDF No #761005, indicating excellent agreement and suggesting the formation of an orthorhombic phase within the *Pc*2_1_*n* group. The refined lattice parameters, with a value of *a* = 8.7430 Å, *b* = 9.3860 Å, and *c* = 5.0795 Å, closely align with the literature reports.^28−31^ The inset of Figure 1a shows a visualization of the crystal structure of GFO. All other simulated parameters are summarized in Table S1. The goodness of fit χ^2^ ∼ 1.42 obtained in refinements, alongside the other fitting parameters such as full width at half-maximum (fwhm) parameters (U, V, and W), supports the favorable acceptance of the fitting parameters.

Figure 1b The SEM image of the pristine electrode displays particle agglomeration, with microsized particles clearly visible. Figure 1 (second row) shows the EDS elemental mapping of the GFO electrode, which confirms the uniform distribution of all elements in the structure. Additionally, Figure S1 illustrates the EDX spectrum of the GFO electrode, clearly indicating the presence of C and GFO elements (Ga, Fe, and O) as evidenced by their respective characteristic X-ray emission lines [C: Kα1,2 (0.277 keV), O: Kα1,2 (0.5249 keV), Fe: Lα1,2 (0.7048 keV), Fe: Kα1,2 (6.4006 keV), Fe: Kβ1,3 (7.0563 keV), and Ga: Lα1,2 (1.093 keV), Kα1,2 (9.24 keV)].

**Figure 2:**
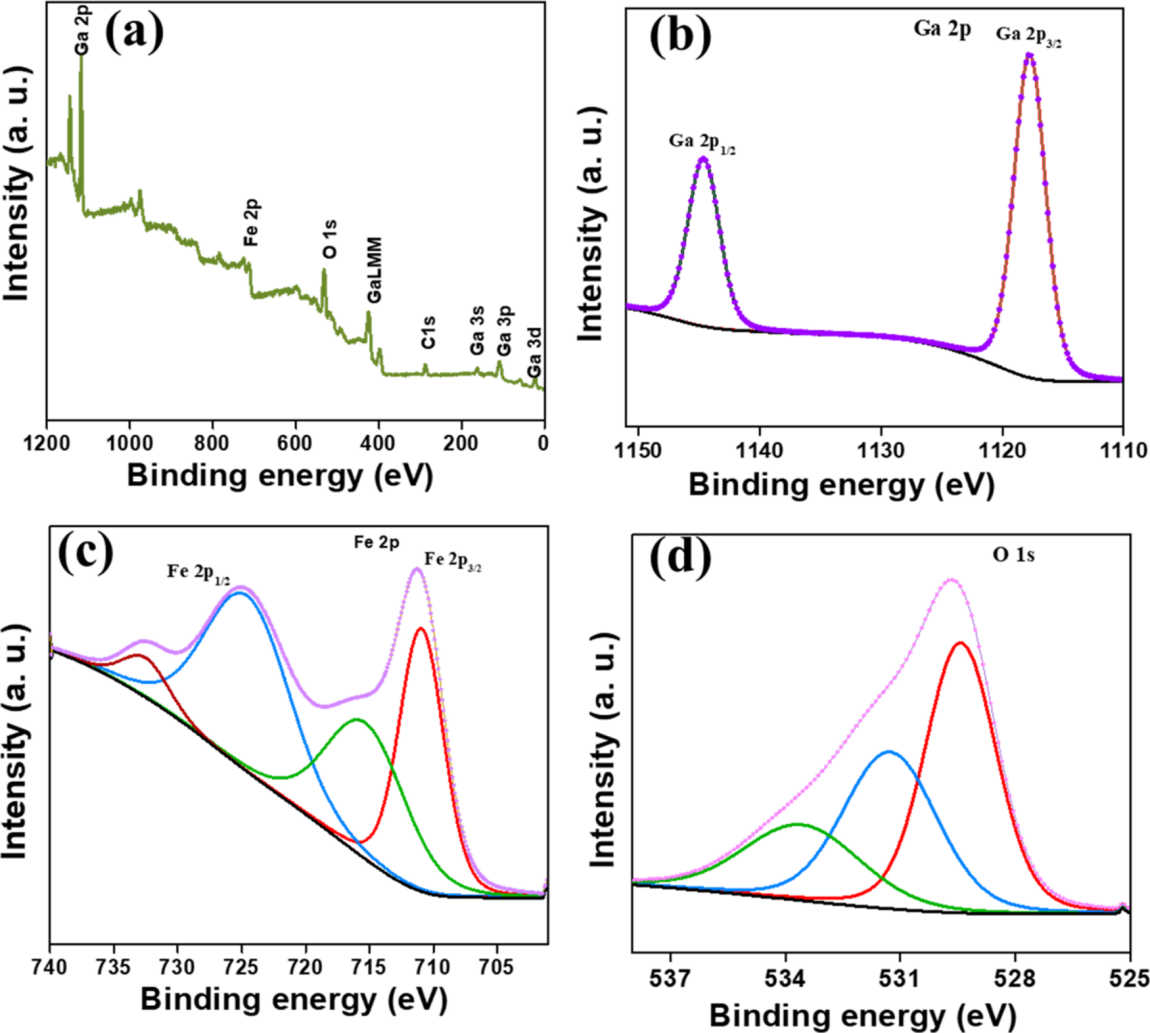
XPS spectra of GFO (a) survey scan and XPS spectra of the (b) Ga 2p, (c) Fe 2p, and (d) O 1s region.

Further details about the oxidation states of the prepared GFO material were obtained through X-ray photoelectron spectroscopy (XPS) studies. Table 1 shows the binding energy values of Ga, Fe, and O elements in the GFO sample. Figure 2a displays the survey spectra of GFO and confirms the presence of Ga 2p, Fe 2p, O 1s, Ga 3s, Ga 3p, Ga 3d, and C 1s (background) peaks, respectively. As depicted in Figure 2b, the Ga 2p spectrum of GFO exhibits two spin–orbit doublets originating from Ga 2p_3/2_ (1117.66 eV) and Ga 2p_1/2_ (1144.65 eV). The binding energy separation between Ga 2p_1/2_ and Ga 2p_3/2_ is approximately ∼ 26.9 eV, indicating the trivalent oxidation state of Ga in GFO^32^ Similarly, in the Fe 2p spectrum of GFO, two distinct peaks at ∼ 725 and ∼ 710 eV are observed, which correspond to the Fe 2p_3/2_ and Fe 2p_1/2_ spin–orbit splitting,^33^ respectively, associated with satellite peaks (Figure 2c). The binding energy difference of ∼ 13 eV between the 2p_3/2_ and 2p_1/2_ peaks confirms the presence of Fe^3+^ in GFO.^34^ Furthermore, the O 1s spectrum of GFO in Figure 2d shows three distinct peaks at ∼ 530, ∼ 531, and ∼ 533 eV, corresponding to metal–oxygen bonds, surface hydroxyl groups, and adsorbed water groups, respectively.^35−37^

**Table 1:** XPS Binding Energy for GFO

GFO	Ga 2p	Fe 2p	O 1s
	Ga 2p_3/2_ (1117.66 eV)	Fe 2p_3/2_ (725.0 eV)	O_1_ (529.39 eV)
	Ga 2p_1/2_ (1144.65 eV)	Fe 2p_1/2_ (710.1 eV)	O_2_ (531.31 eV)
			O_3_ (533.67 eV)

**Figure 3:**
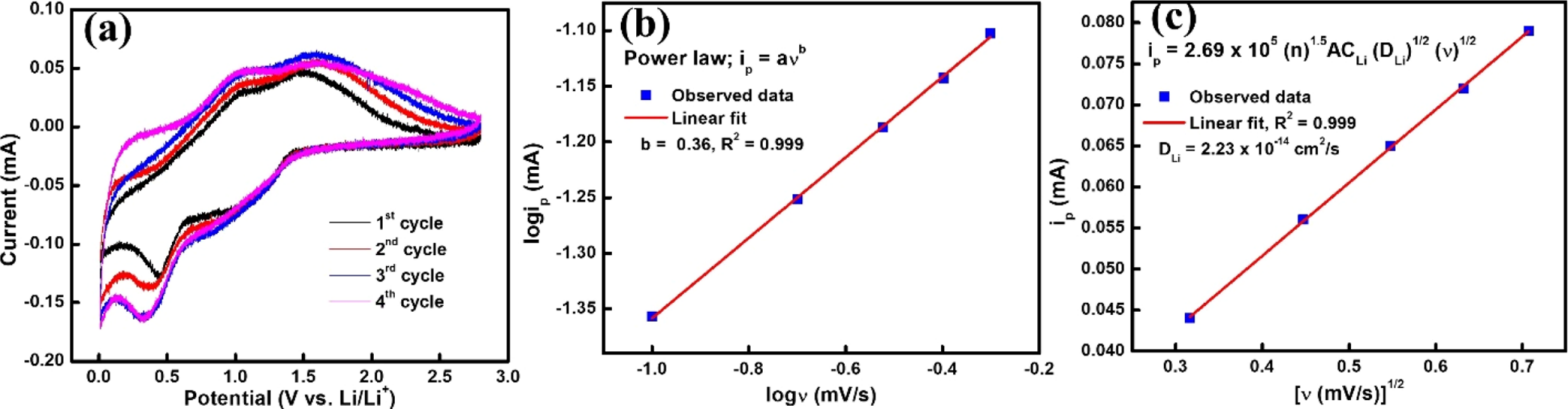
(a) CV curves at 0.1 mV s^–1^ scan rate (Initial four cycles), (b) logarithm plot of peak current (*i*_p_) vs scan rate (*ν*), and (c) plot *i*_p_ vs *v*^1/2^ for GFO.

Figure 3a presents the cyclic voltammograms (CV) of the GFO electrode at a scan rate of 0.1 mV s^–1^ between the 0.01 and 2.8 V voltage window (vs Li/Li^+^). The CV shows that the redox peak of the first cycle is notably distinct from that of the subsequent cycles, and there are no noticeable changes in the redox peaks in the third and fourth cycles. The reversible cathodic peak at ∼0.4 V observed during the first discharge cycle is attributed to the formation of solid-electrolyte interphase (SEI) film and the reduction of GaFeO_3_ to Fe and Ga through lithium insertion, along with the formation of noncrystalline Li_2_O (eq 1).^38^ In the subsequent cycle of anode scanning, an oxidation peak is observed at around 1 V. These peaks can be attributed to the delithiation of Li_2_Ga and the reduction of Fe^2+^ to Fe (eqs 2 and 3).^38,39^ Based on the CV results, the conversion-alloy reaction mechanism can be expressed in the following (eqs 1–3).
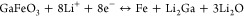
1
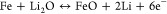
2
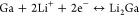
3The multiscan CV was conducted to explore the electrochemical lithium storage performance and kinetics of the GFO. Figure S2a shows the CV curve of the electrode material at a scan rate of 0.1–0.5 mV s^–1^, where the relationship between the scan rate (*v*) and peak current (*i*_p_) can be expressed as given in eq 4.^40^
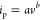
4Which can be rewritten as eq 5,
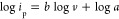
5Where *a* and *b* are adjustable parameters, and the slope of the plot log(*v*) vs log(*i*_p_) (Figure 3b) determines the value of b. A value of *b* = 0.5 suggests that the electrode’s storage behavior is governed by the diffusion phenomenon, while *b* = 1 indicates that the electrode’s storage behavior resembles capacitive behavior, which is controlled by the adsorption process^41^ The calculated value of *b* is 0.36 from anodic peak currents, indicating that the energy storage behavior in GFO electrodes is a diffusion-controlled process. Similar phenomena were observed in the literature.^42^ The quantitative value of diffusion coefficients (*D*_Li_) was determined using the Randles–Sevcik eq 6, where *i*_p_ (A) is related to the square root of *v* as follows eq 6.
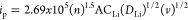
6Where *n* represents the number of electrons participating in the electrode reaction (*n* = 2 for GFO), CLi (1.0 × 10^–3^ mol cm^–3^ for 1 M LiPF_6_) denotes the lithium ions’ concentration, *ν* indicates the scan rate (mV s^–1^) of the cyclic voltammetry, *A* (0.785 cm^2^) corresponds to the contact area of the electrode, and 2.69 × 10^5^ (C mol^–1^ V^–1^) is a constant factor. The value of *D*_Li_ in the GFO anode, which is depicted in (Figure 3c), closely aligns with the literature.^43^ In addition, the CV measurements, depicted in Figure S2b, were consistently performed at a scan rate of 0.1 mV s^–1^ after 250 cycles, indicating excellent electrochemical reversibility and stability and highlighting the reliability of the electrochemical processes.

**Figure 4:**
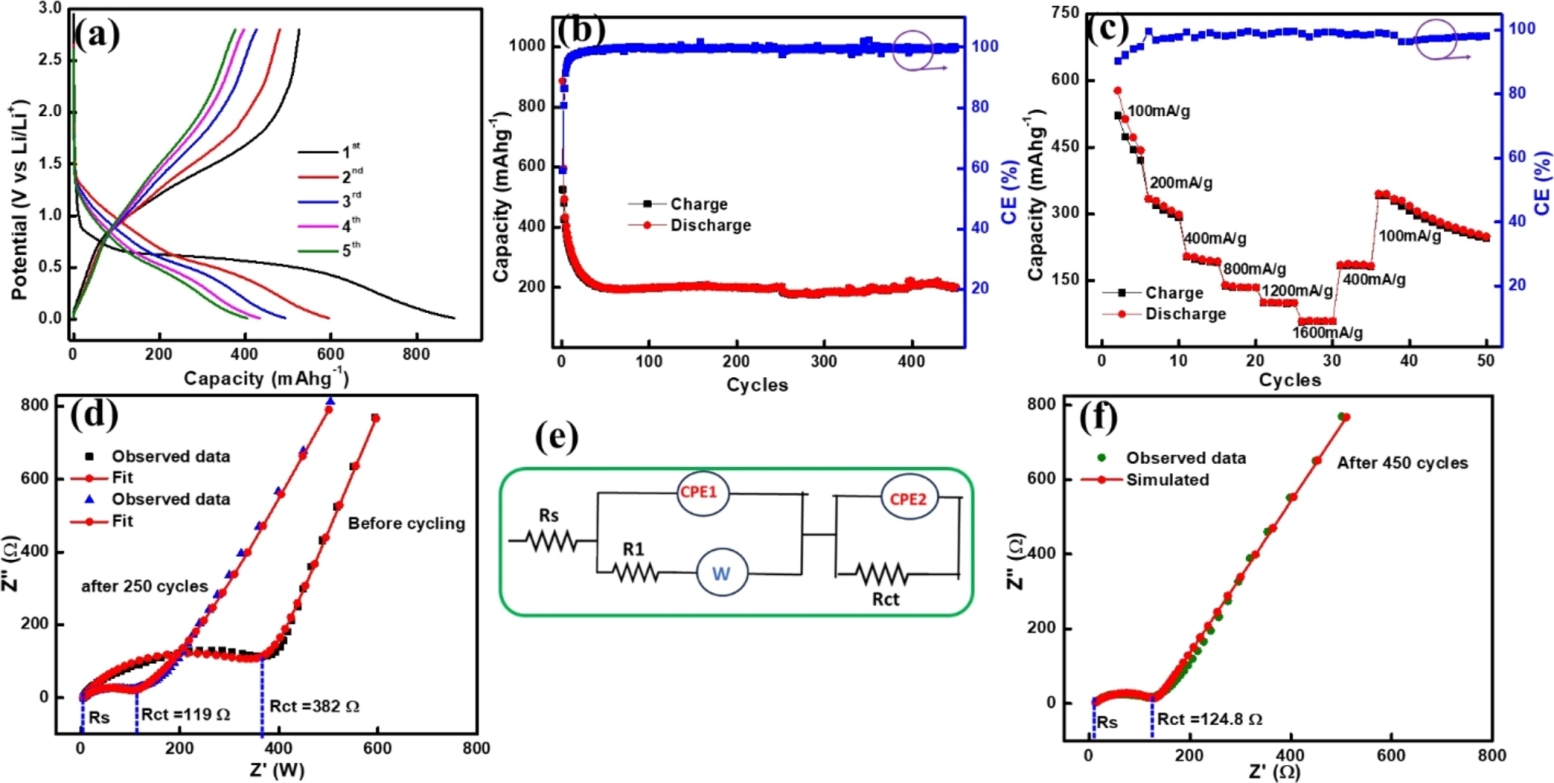
(a–c) Charge–discharge profile, capacity vs cycles, and rate capability (2nd −50 cycles) for GFO, (d) complex plane plots of before cycling and after 250 cycles, (e) equivalent circuit model for EIS fitting, and (f) complex plane plots of after 450 cycles.

Further, galvanostatic charge–discharge (GCD) cycle tests were conducted to assess the cycling performance of the GFO electrodes. Figure 4a shows the capacity versus voltage profiles of the GFO electrode at a current density of 100 mA g^–1^ in the potential range of 0.01–2.8 V. The first discharge curve is composed of a plateau below 1 V, which might correspond to the conversion of Fe^3+^ to Fe^0^ and Ga^3+^ to Ga^0^ in the electrode during the initial Li insertion. The GFO sample exhibits initial charge and discharge capacities of 891 and 527 mA h g^–1^, respectively. The first cycle irreversible capacity corresponds to a CE of 59 %, attributed to the decomposition of the electrolyte resulting in the formation of a stable SEI on the GFO electrode surface. After the first cycle, the columbic efficiency gradually increases and reaches 94 % by the fifth cycle. At the end of 450 cycles, Coulombic efficiency is 99.6 %. The cyclic stability, measured at the current density of 100 mA g^–1^ on the GFO electrode, demonstrates a capacity retention of ∼ 200 mA h g^–1^ with a CE of 99.6 % over 450 cycles (Figure 4b). Notably, these results are comparable with the recent findings on similar types of materials.^38,44,45^

The rate capability test was carried out for the GFO electrode at different current rates. Figure 4c shows that it delivers discharge capacities of 335, 205, 141, 100, and 57 mA h g^–1^ at current densities of 200, 400, 800, 1200, and 1600 mA g^–1^ rates, respectively. The battery retained its original capacity when the current was reduced to 400 and 100 mA h g^–1^.

Moreover, EIS measurements were performed to assess the electrical properties of the electrochemical systems of the GFO anode. EIS is widely utilized in battery research, corrosion studies, sensor development, etc., due to its nondestructive nature. It provides crucial insights into the interface between electrodes and electrolytes and offers valuable information about the performance and stability of electrochemical devices. The EIS data recorded for GFO anodes were simulated using different resistance, capacitance, and constant phase element elements (CPE). The model that best fits (Figure 4e) the experimental data was chosen based on its closest alignment with the recorded data. Complex plane plots,^46^ depicting real impedance on the *x*-axis and imaginary impedance on the *y*-axis, were generated for both pre and postcycling conditions, as illustrated in Figure 4d,f.

In the equivalent circuit model, *R*_s_*R*_1_, and *R*_ct_ represent the solution resistance, SEI resistance, and charge transfer resistance, respectively.^47^*R*_s_ typically manifests as a semicircular arc in the high-frequency region of the complex plane plot, symbolizing the resistance encountered within the electrolyte solution in the electrochemical cell. On the other hand, *R*_ct_ is commonly depicted by a semicircular arc in the midfrequency region of such a plot, symbolizing the resistance associated with charge transfer processes occurring at the electrode–electrolyte interface. This resistance reflects the ease or difficulty with which charge carriers, such as ions or electrons, can transfer across this interface. Thoroughly analyzing these parameters is crucial, as they delineate the performance and stability of batteries. The simulated EIS results are given in Table 2. Before cycling, the *R*_s_ value was 4.4 Ω, which was almost equal even after 250 cycles: however, it slightly increased (10.4 Ω) after 450 cycles. On the other hand, the *R*_ct_ value of cells was significantly reduced from 382 Ω (before cycling) to 119 Ω (after 250 cycles). The reduced value of *R*_ct_ after cycling facilitates faster kinetics and stability of the battery for longevity.

**Table 2:** EIS Fitting Parameters for GFO Electrodes

	conditions		
parameters	(before cycling)	(250 cycles)	(450 cycles)
*R*_s_ (Ω)	4.4	4.2	10.4
CPE1 (Fs^(n1–1)^)	8.4 × 10^–4^	3.9 × 10^–3^	6.0 × 10^–3^
*W* (Ωs^–1/2^)	1.1 × 10^–4^	1.3 × 10^–18^	3.3 × 10^–16^
CPE2 (Fs^(n2–1)^)	4.6 × 10^–5^	1.0 × 10^–5^	8.4 × 10^–5^
*n*_2_	0.68	0.54	0.52
*R*_ct_ (Ω)	382	119	124.8
χ^2^ (10^–3^)	3.01	5.06	2.58

In addition, the incorporation of CPE into the circuit model is indispensable as the interface does not exhibit an ideal capacitor^48^ The CPE’s impedance, denoted as *Z*_CPE_, adheres to a power-law relationship^40,49^ (eq 7).
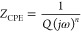
7Where *Q* (Ω^–1^s*^n^*) denotes the constant associated with the CPE, a more precise unit of representation is Fs^*n*–1^, which describes capacitive behavior^50^ The symbol *j*(√−1) is the imaginary unit ω (=2π*f*), signifies the angular frequency, and *n* is the dimensionless constant. When *n* is −1, the CPE demonstrates inductive behavior; *n* = 1, the CPE behaves as a pure capacitor, and for *n* equal to 0.5, the CPE is similar to the Warburg element (*W*).

*W* is a significant parameter related to lower frequencies employed to represent diffusion-controlled processes within the electrode or electrolyte of an electrochemical system. The impedance of the Warburg element (*Z*_W_) varies with frequency and is typically expressed by an equation such as eq 8
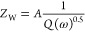
8In this equation, *A* denotes the proportionality constant. The relatively small magnitude of χ^2^ ∼ 10^–3^, coupled with its favorable alignment with the other optimized parameters of the equivalent circuit model, robustly corroborates the appropriateness of this model as the optimal fit.

**Figure 5:**
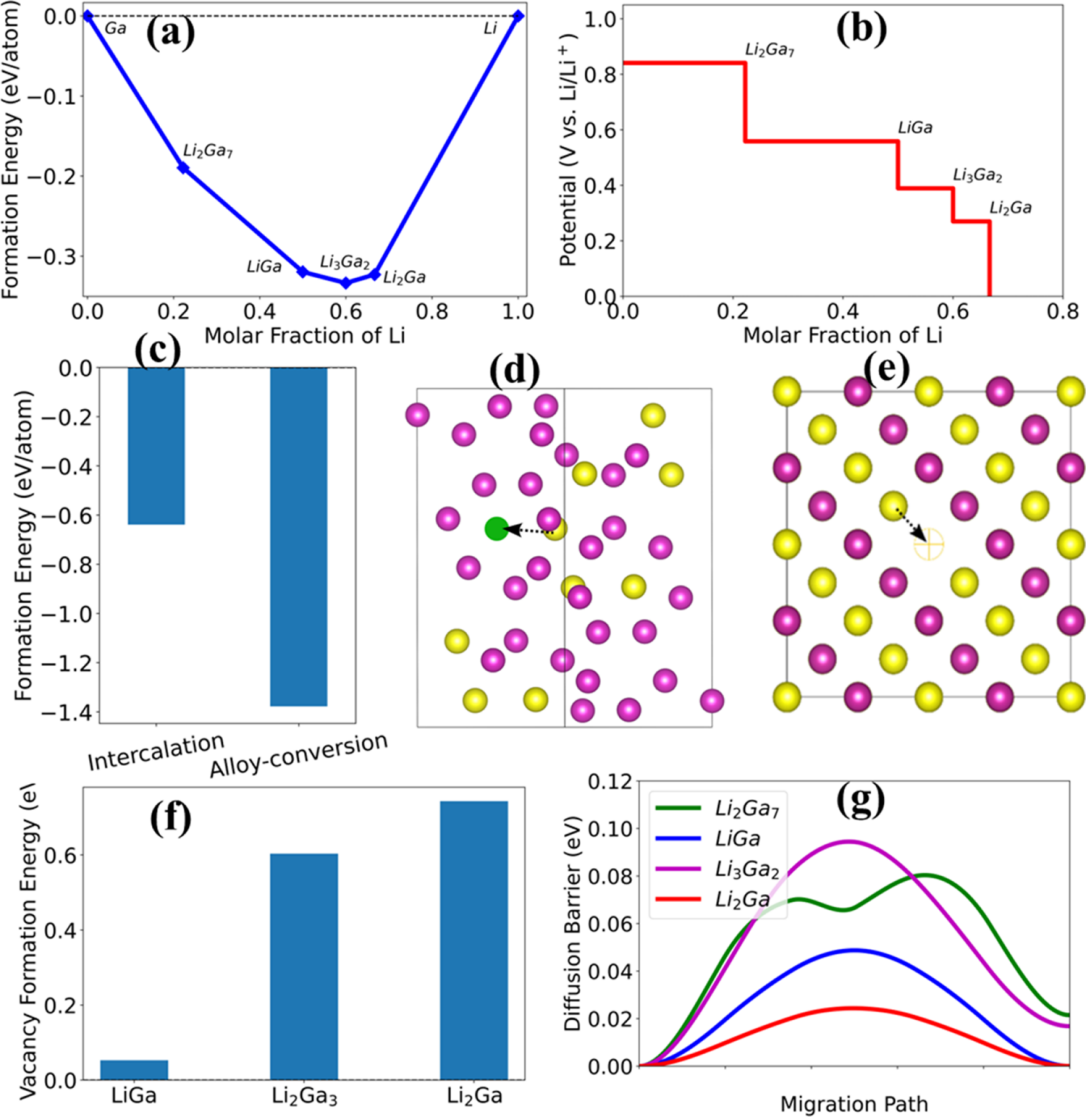
(a) Energy convex hull diagram of Li-Ga alloys (b) calculated electrochemical potentials of Ga alloying with Li-ions in LIBs, (c) formation energy comparison of intercalation products and alloy-conversion products of GFO anode, (d) structure of Li_2_Ga_7_, green sphere represents the interstitial space available for Li migration, (e) Li-ion conduction mechanism in LiGa alloy (the empty circle represents the vacant site and the arrow shows the diffusion path of Li-atom to the vacant site), (f) vacancy formation energy in Li-Ga alloys, and (g) diffusion barrier potential for Li to diffuse to the unoccupied site in Li-Ga alloys.

DFT calculations were performed to investigate the electrochemical behavior of the GFO anode in LIBs. The thermodynamically stable composition of Li and Ga was identified, and a convex hull diagram was created. This is crucial for understanding the reaction mechanism and the reaction pathways of electrode materials in LIBs. The convex hull diagram of the Ga-Li system is presented in Figure S3a. DFT-calculated convex hull analysis indicates the formation of Li_2_Ga_7_ alloy at lower lithium (Li) concentration, followed by the formation of LiGa alloy at intermediate concentration, and subsequently Li_3_Ga_2_ and Li_2_Ga at higher Li concentrations. Table S2 summarizes the calculated lattice parameters of GFO, Li intercalated Li_0.125_GaFeO_3_, and Li-Ga alloys. The results of convex hull analysis agree well with the experimental results of the alloying conversion reaction of the GFO anode in LIBs. A similar reaction mechanism was reported in the previous studies.^51−53^Figure 5b presents the calculated electrochemical potentials of Ga alloying with Li-ions as a function of Li fraction. The electrochemical potential can be approximated using^54−56^eq 9.
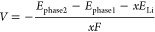
9where *x* represents the number of Li ions required to transition from phase 1 into phase 2. *E*_(phase)_ and *E*_Li_ are the DFT-calculated energies of Li-Ga alloys and Li metal, respectively.

The electrochemical potential of about 0.84 V was approximated to that of the corresponding Li_2_Ga_7_ alloy. The next potential of about 0.55 V was calculated, corresponding to the transition of Li_2_Ga_7_ into LiGa. Similarly, potentials of about 0.38 and 0.27 V were calculated, corresponding to the transition of LiGa to Li_3_Ga_2_ and Li_3_Ga_2_ to Li_2_Ga, respectively. Similar values of the electrochemical potential of Ga were reported in previous studies.^52,57,58^ To investigate the possibility of the intercalation mechanism of the GFO anode, we compared the calculated formation energy of intercalation products vs alloy-conversion products. The Li intercalated structure of Li_0.125_GaFeO_3_ can be found in Supporting Figure S3a. A properly balanced equation between intercalation products and alloy-conversion products was derived from eq 8, which can be simplified as eq 10,
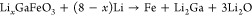
10Where *x* represents a fraction of Li intercalated in GaFeO_3_. Formation energy can be calculated using the following eq 11,
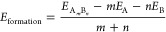
11where *E*_A*_m_*B*_n_*_, *E*_A_, and *E*_B_ represent the DFT-calculated energy of compound A*_m_*B*_n_*, A, and B, respectively.

Figure 5c compares and presents the total formation energy of the intercalation products and alloy-conversion products. The formation energy of alloy-conversion products was predicted to be relatively smaller than that of intercalation products. The formation energy comparison clearly points to the likelihood of an alloy conversation mechanism rather than an intercalation mechanism in the GFO anode (Figure 5a,b).

To understand the transport kinetics of Li ions in Li-Ga alloy, we calculated diffusion barrier potential and vacancy formation energy. Li_2_Ga_7_ crystallizes in a rhombohedral lattice system with the *R*3̅*m* space group.^51^ The open crystal structure and large interstitial spaces of Li_2_Ga_7_ can provide pathways for Li-ions to travel within the material. Figure 5d illustrates the structure of Li_2_Ga_7_; the green sphere represents the interstitial space available for Li migration. The vacancy can also act as a pathway for Li-ion transport within the material, enhancing its conductivity and leading to improved electrochemical performance.^59,60^ To gain insight into the effects of vacancies, supercell structures were modeled, and vacancy formation energy was calculated. Figure 5e depicts a schematic representation of Li-vacancy in LiGa alloy, with an arrow indicating the path of Li diffusion to the unoccupied site; the structures illustrating Li vacancy and migration path for Li_3_Ga_2_ and Li_2_Ga can be found in Supporting Figure S3b,c respectively. Figure 5f illustrates the vacancy formation energy in Li-Ga alloys, and Figure 5g shows the Li diffusion barrier potential across the migration path. The Li diffusion barrier potential was predicted to be about 80, 50, 95, and 25meV, corresponding to Li_2_Ga_7_, LiGa, Li_3_Ga_2_, and Li_2_Ga, respectively. The vacancy formation energies in Li_3_Ga_2_ and Li_2_Ga were calculated to be relatively higher, indicating less likelihood of vacancy formation in these alloys. On the other hand, because of the smaller vacancy formation energy and lower barrier potential of LiGa alloy, in addition to Li_2_Ga_7_, it could play an important role in enhancing the Li diffusion during the alloy-conversion mechanism in GFO anode material.

## Conclusions

In summary, we have prepared an alloy-conversion-based GFO anode to address the volume expansion and conversion issues observed in both pure alloy and conversion-based anodes. The crystallographic phase of the material prepared by the one-step solid-state method, as inferred from the powder X-ray diffraction, is in the pure perovskite phase with an orthorhombic structure. The electrochemical properties of GFO, used as an anode material in LIBs and tested in a Li half-cell configuration, demonstrate an initial discharge capacity of ∼ 887 mA h g^–1^ at a current density of 100 mA g^–1^. After 450 cycles, it retains a capacity of ∼ 200 mA h g^–1^. Additionally, it exhibits a high-rate capability at a current of 1600 mA g^–1^. Cyclic voltammetry studies further indicated that the alloy-conversion-based reaction mechanism is occurring in the GFO anode. Furthermore, DFT studies confirm that Li_2_Ga_7_ and LiGa could significantly impact the ease of Li-ion migration through the crystal lattice, thus, enhancing Li-ion mobility within the structure. These promising results suggest that the untapped perovskite-based GFO anodes could be future potential candidates for LIBs application and potentially serve as substitutes for alloy/conversion-based materials.

## Supporting Information Available

Rietveld refined parameters and agreement factors for GFO at room temperature with Pc2_1_n space group (Table S1); DFT-calculated lattice parameters of GFO, Li intercalated Li_0.125_GaFeO_3_, and Li-Ga alloys (Table S2); EDS spectra of GFO electrode (Figure S1); (a) multiscan CV curves at various scan rates from 0.1 to 0.5 mV s^–1^ before cycling and (b) CV curve recorded after 200 cycles at 0.1mV s^–1^ for GFO electrode (Figure S2); (a) Li intercalated structure of Li_0.125_GaFeO_3_ (b, c) the structure of Li_3_Ga_2_ and Li_2_Ga, respectively (Figure S3); ex-situ SEM images (a, b) discharged and (c, d) charged GFO electrodes (Figure S4) (PDF)
