# Supplementary material for: Unveiling Potential of Gallium Ferrite (GaFeO3) as an Anode Material for Lithium-Ion Batteries
Source: ACS Omega. 2024 Sep 13;9(38):39863–72. doi: 10.1021/acsomega.4c05437 (PMC11425807; doi:10.1021/acsomega.4c05437)
Supplement: Supplementary file 1 — ao4c05437_si_001.pdf [file ao4c05437_si_001.pdf]

Supporting Information

Unveiling Potential of Gallium Ferrite ($GaFeO_3$) as an Anode Material for Lithium-Ion Batteries

Mohan K Bhattarai[1*], Shweta Shweta[1], Moses D Ashie[2], Shivaraju Guddehalli Chandrappa[1*], Birendra Ale Magar[3], Bishnu P Bastakoti[2], Ubaldo M Córdova Figueroa[3], Ram S Katiyar[1], Brad R Weiner[4], and Gerardo Morell[1]

[1]Department of Physics, University of Puerto Rico, San Juan, PR, 00931, USA
[2]Department of Chemistry, North Carolina A&T State University, 1601 East Market Street, Greensboro, NC 27411, USA

[3]Department of Chemical Engineering, University of Puerto Rico - Mayagüez, PR, 00681, USA
[4]Department of Chemistry, University of Puerto Rico, San Juan, PR, 00931, USA

*Correspondence author's email: mohankbhattarai@gmail.com and shivugc123@gmail.com

Table S1: Rietveld refined parameters and agreement factors for GFO at room temperature with Pc21n space group.

Sample	Parameters								
	a (Å)	b (Å)	c (Å)	U	V	W	R_{wp} (%)	R_{exp} (%)	$\chi2$
GFO	8.7430	9.3860	5.0795	0.00549	-0.01578	0.05769	17.1	14.3	1.42

Table S2: DFT calculated lattice parameters of GFO, Li intercalated $Li_{0.125}GaFeO_3$ and Li-Ga alloys.

Structure	Calculated values			Literature values			Reference
	a (Å)	b (Å)	c (Å)	a (Å)	b (Å)	c (Å)	
GFO ($GaFeO_3$)	8.843	9.509	5.112	8.751	9.399	5.080	[1]
$Li_{0.125}GaFeO_3$	8.842	9.566	5.172				
Li_2Ga_7	8.514	8.514	16.981	8.441	8.441	16.793	[2]
LiGa	6.253	6.253	6.253	6.177	6.177	6.177	[2]
Li_3Ga_2	4.379	4.379	13.819	4.367	4.367	13.896	[3]
Li_2Ga	4.544	9.482	4.339	4.56	9.54	4.36	[2]

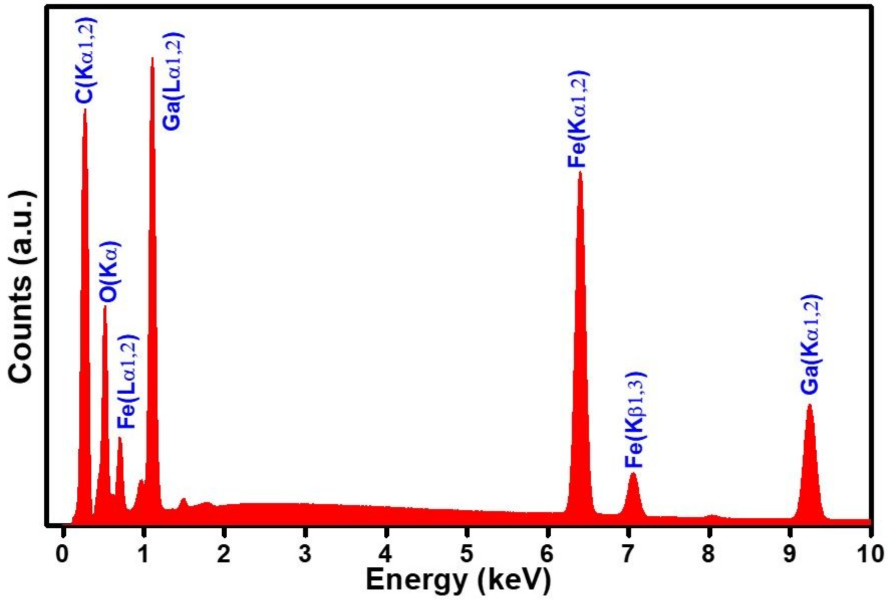

Figure: S1 EDS spectra of GFO electrode.

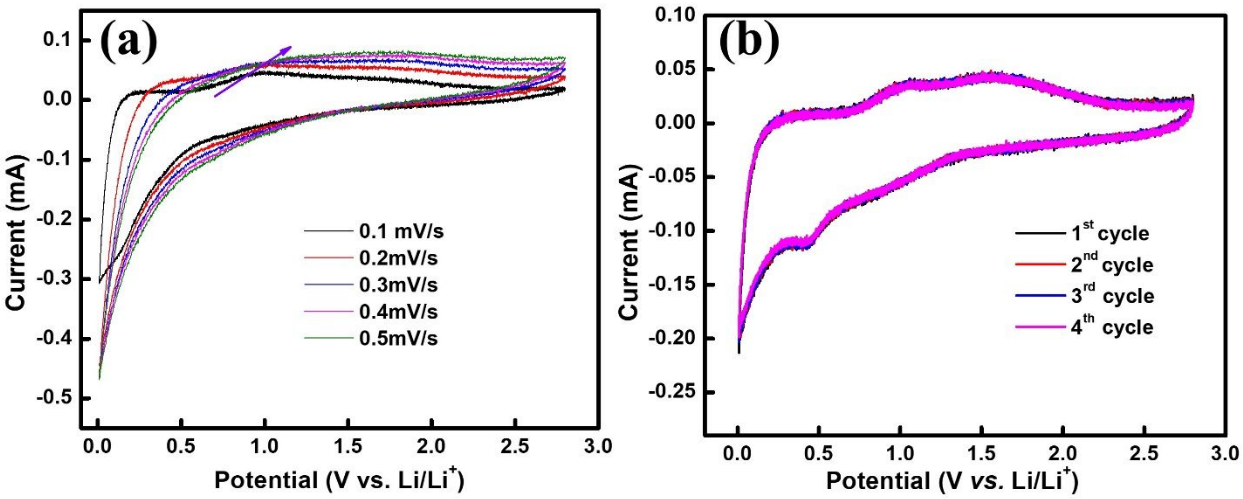

Figure S2 (a) multi-scan CV curves at various scan rates from 0.1 to 0.5 mV/s before cycling **(b)** CV curve recorded after 250 cycles at 0.1mVs for GFO/C

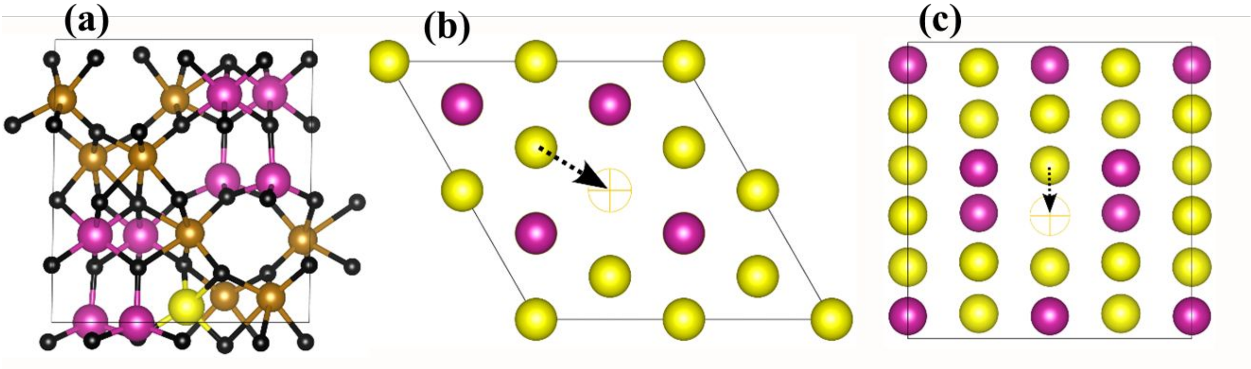

Figure S3: (a) Li intercalated structure of $Li_{0.125}GaFeO_3$ (b & c) the structure of Li_3Ga_2 and Li_2Ga, respectively (the empty circles represent the Li-vacant site, and the arrow shows the migration path for Li to diffuse).

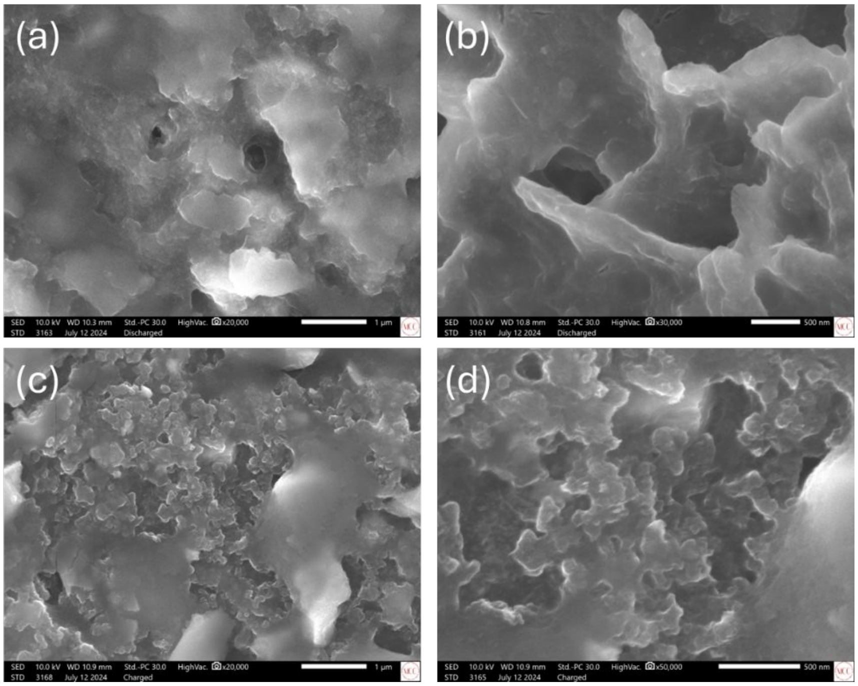

Figure S4 Ex-situ SEM images (a-b) discharged and (c-d) charged GFO electrodes.

References:

(1) Abrahams, S. C.; Reddy, J. M.; Bernstein, J. L. Crystal Structure of Piezoelectric Ferromagnetic Gallium Iron Oxide. *J Chem Phys* **1965**, *42* (11), 3957–3968. https://doi.org/10.1063/1.1695868.

(2) Saint, J.; Morcrette M.; Larcher D.; Tarascon J.M. Exploring the Li-Ga Room Temperature Phase Diagram and the Electrochemical Performances of the Li_xGa_y Alloys vs. Li. *Solid State Ion* **2005**, *176* (1–2), 189–197. https://doi.org/10.1016/j.ssi.2004.05.021.

(3) Hafner, J.; Jank, W. Structural and Electronic Properties of Crystalline and Molten Zintl Phases: The Li-Ga System. *Phys Rev B* **1991**, *44* (21), 11662–11676. https://doi.org/10.1103/PhysRevB.44.11662.
